# STED super-resolution microscopy unveils the dynamics of Atg30 on yeast Pex3-labeled peroxisomes

**DOI:** 10.1016/j.isci.2024.110481

**Published:** 2024-07-08

**Authors:** Eline M.F. de Lange, Frank N. Mol, Ida J. van der Klei, Rifka Vlijm

**Affiliations:** 1 Molecular Cell Biology, Groningen Biomolecular Sciences and Biotechnology Institute, University of Groningen, Nijenborgh 7, 9747 AG Groningen, the Netherlands; 2 Molecular Biophysics, Zernike Institute for Advanced Materials, University of Groningen, Nijenborgh 4, 9747 AG Groningen, the Netherlands

**Keywords:** Biological sciences, Molecular biology, Structural biology, Resolution techniques

## Abstract

Peroxisomes are dynamic organelles with important metabolic functions. Yeast Pex3 is a multifunctional membrane protein aiding in peroxisomal biogenesis, inheritance, and degradation (pexophagy), by interacting with process-specific factors. Using multicolor (live-cell) stimulated emission depletion (STED) nanoscopy, we studied the localization of Pex3 and its binding partners in *Hansenula polymorpha.* Unlike confocal microscopy, STED allows resolving the membrane of tiny peroxisomes, enabling accurate measurements of the size of all Pex3-labeled peroxisomes. We localized Pex3 and its binding partners at peroxisome-repressing and -inducing conditions and during pexophagy. In-depth quantitative analysis of Pex3 and pexophagy receptor Atg30 showed dynamic changes in their (co)localization. One remarkable response of Atg30 was the shift in position from being sandwiched between clustered peroxisomes at proliferation conditions, to the cytosolically exposed parts of peroxisome clusters upon pexophagy induction. Summarizing, we show that STED allows characterizing dynamics of the localization of peroxisomal proteins in yeast cells.

## Highlights

• STED nanoscopy is feasible in living yeast cells
• STED allows characterizing the dynamics of peroxisomal proteins in yeast
• Changed colocalization of Pex3 and its binding partners is accurately quantified
• Atg30 relocates on the peroxisomal membrane upon pexophagy induction

## Graphical abstract

**Figure:**
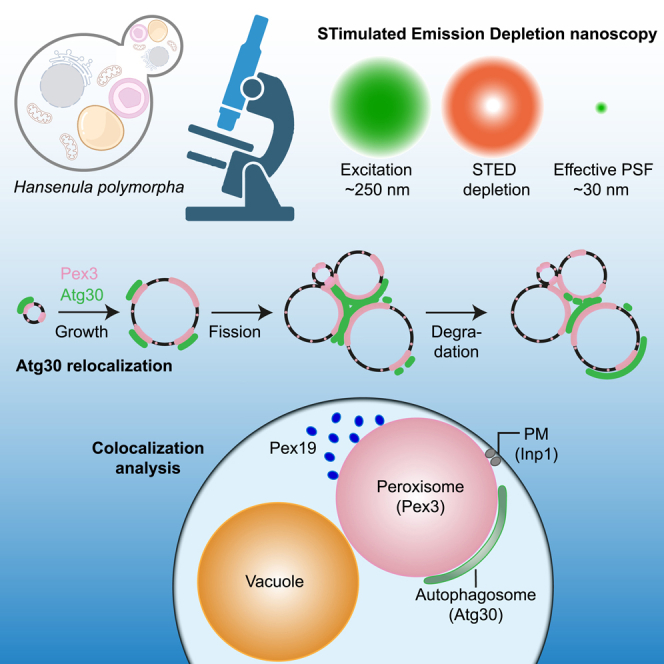

## Introduction

Peroxisomes are crucial, multifunctional organelles that are present in most eukaryotic cells.^1^^,^^2^ Common metabolic functions include hydrogen peroxide and lipid metabolism. In response to environmental changes, these organelles are dynamic in function, size, and abundance, while balancing their growth, division, and selective degradation by autophagy (pexophagy).^3^^,^^4^ In these processes, proteins involved in peroxisome biogenesis and proliferation (encoded by *PEX* genes) perform many different functions.^5^^,^^6^

Pex3 is a key protein in peroxisome biology.^7^ Thus far, several roles of this integral peroxisomal membrane protein (PMP) have been described. Pex3 is required for sorting of newly synthesized PMPs together with the cytosolic chaperone/receptor Pex19.^8^^,^^9^^,^^10^ Next to this, Pex3 is indispensable for the retention of peroxisomes in yeast mother cells, by binding inheritance protein 1 (Inp1).^11^ Inp1 associates with the plasma membrane (PM),^12^^,^^13^ thereby anchoring the peroxisome. Pex3 is also required for the formation of peroxisome-vacuole contact sites, whereby Pex3 accumulates in relatively large patches at this membrane contact site.^14^ A Pex3 binding partner on the vacuole has not been identified yet. Pex3 is also involved in pexophagy.^15^^,^^16^^,^^17^

The methylotrophic yeast species *Hansenula polymorpha* and *Pichia pastoris* are very attractive organisms to study pexophagy. In these organisms, peroxisomes play a key role in methanol (MeOH) metabolism.^18^ Both yeast species are not only ideal model organisms in fundamental peroxisomal research but also used for peroxisome engineering aiming at applications in industrial biotechnology.^19^^,^^20^^,^^21^ During growth on MeOH, multiple large peroxisomes are formed that contain key enzymes of MeOH metabolism. Upon transferring these cells to glucose- or ethanol-containing media, these enzymes are no longer needed, and the whole organelle is selectively degraded by pexophagy. In *H. polymorpha*, this invariably occurs via macropexophagy, whereas in *P. pastoris* peroxisomes are degraded by either micropexophagy (upon a shift to glucose medium) or macropexophagy (upon a shift to ethanol medium).^22^^,^^23^

During macropexophagy, peroxisomes are one by one secluded from the cytosol by several autophagosomal membrane layers, which fuse with the vacuole.^15^^,^^24^ Pex3 is essential for this process, because it recruits and interacts with a pexophagy-specific receptor, Atg30. Much is known about the role of *Pp*Atg30, which associates with the adaptor protein Atg11, a crucial step to deliver peroxisomes to the pre-autophagosomal structure.^25^^,^^26^

The function of *Pp*Atg30 is regulated by posttranslational modifications. *Pp*Atg30 is massively phosphorylated by Hrr25 (casein kinase 1δ) upon induction of pexophagy. *Pp*Atg30 phosphorylation is negatively regulated by Pex3 and positively by Atg37 (a PMP involved in the assembly of the pexophagy receptor protein complex), which compete to bind Atg30.^27^^,^^28^

*H. polymorpha* contains a homologue of *Pp*Atg30. Although *Hp*Atg30 has not been studied in detail yet, it was identified as a binding partner of *Hp*Pex3.^29^ *Saccharomyces cerevisiae* Pex3 also bind a pexophagy-specific receptor, *Sc*Atg36. *Sc*Atg36 has a similar function as *Pp*Atg30 but shows no homology with this protein.^17^^,^^30^ Atg30/36 is involved in pexophagy and thus expected to be mainly phosphorylated when peroxisomes are no longer required for growth (e.g., upon a shift of cells to media containing glucose).

Although much is known about the different roles of Pex3 in the above-mentioned processes, how and when Pex3 binds to the different identified binding partners (Pex19, Inp1, Atg30/36, and a yet unknown component at the vacuole) remains unrevealed. Interestingly, Pex3 is distributed over the entire peroxisomal surface, but also accumulates at specific regions, for instance at peroxisome-vacuole and peroxisome-PM contact sites.^12^^,^^14^ At peroxisome-PM contacts, Inp1 also accumulates, suggesting that regions with enhanced Pex3 concentrations represent sites where specific Pex3 binding partners localize.

Microscopy studies are indispensable in peroxisome research. However, the resolution limit of traditional fluorescence microscopy (FM) methods is too low to determine the exact peroxisomal numbers and size or to localize proteins of interest precisely. For instance, newly formed peroxisomes are generally too small to identify. Although the resolution of electron microscopy is much better, this technique has several disadvantages. Cells have to be fixed, and Z sections have to be analyzed individually, making it difficult to quantitatively measure the size of each organelle with high data acquisition. To overcome the diffraction-limited resolution in conventional fluorescence microcopy, we here use the super-resolution microscopy technique stimulated emission depletion (STED). We show that even in small peroxisomes, STED allows to resolve the membranes and determine the spatial distribution of specific proteins. So far, STED has been successfully applied in only two studies on peroxisomes.^31^^,^^32^ However, these studies were performed in mammalian cells, not in yeast. These revealed a heterogeneous distribution of specific peroxisomal proteins in human. Also, these studies did not include the localization of Pex3 or Atg30 (which is absent in mammals).

Here, we studied the spatiotemporal distribution of *H. polymorpha* Pex3. To achieve this, we visualized and quantified the distribution and (co)localization of both *Hp*Pex3 and its binding partners at various stages of peroxisome proliferation and pexophagy. We used (live-cell) STED nanoscopy, which allows to accurately quantify the number and size of Pex3-labeled peroxisomes. To visualize the complete cycle of peroxisome repression, induction (proliferation), and degradation by pexophagy with the exact localization of several proteins, we used automated STED imaging, which generated a large dataset of images in each of the stages. The increased resolution due to the STED imaging and the large data acquisition due to automation gave a precise and accurate view on the complex localization of Pex3 and its studied binding partners, thereby especially highlighting the dynamic distribution of Atg30. Our data showed that STED is feasible in yeast peroxisomal research, unveiling that Pex3 specifically clusters with its binding partners, dependent on the stage in the peroxisomal cycle. Of special interest is a shift in the localization of Atg30 upon pexophagy induction, from being sandwiched between clustered peroxisomes during proliferation, to a localization at the cytosolically exposed regions of peroxisome clusters upon pexophagy induction.

## Results

### Validation of STED nanoscopy for yeast peroxisomes

STED has not yet been applied in yeast peroxisomal studies before, so we first investigated the feasibility of this method. To enable labeling with STED-suitable dyes, we fused endogenous Pex3 with either the HaloTag^33^ or SNAP-tag^34^ at the C-terminus. Pex3-GFP, which has been used before to analyze *H. polymorpha* peroxisomes by fluorescence microscopy,^12^^,^^14^ was included as a control. All three strains containing tagged Pex3 variants grew similar on methanol as the WT control, indicating that the tags did not affect peroxisome function (Figure S1).

**Figure 1:**
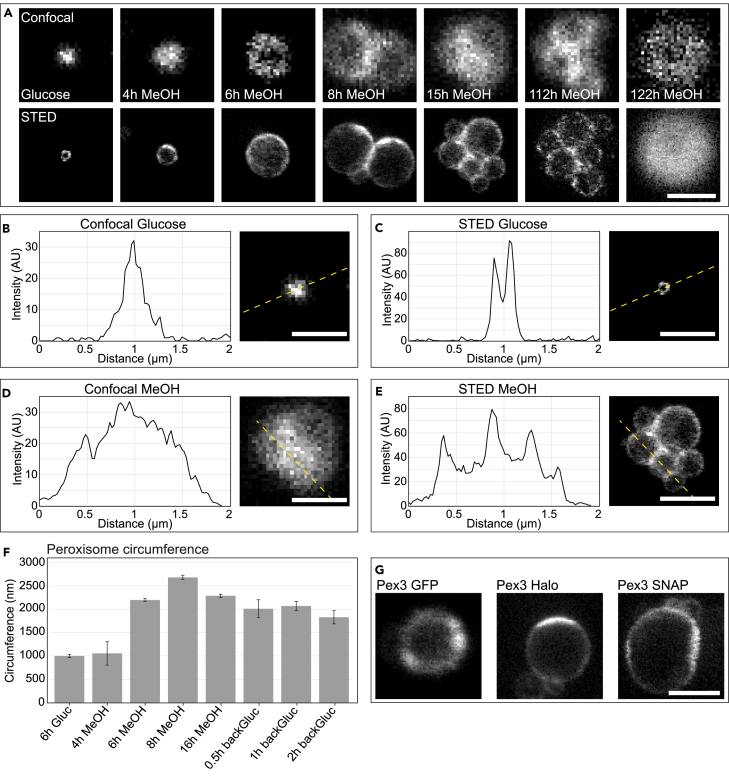
Validation of STED nanoscopy for yeast peroxisome imaging (A) Overview of endogenous Pex3-Halo with SiR labeling in fixed *H. polymorpha* cells, when grown either on glucose-containing medium (left panel) or upon shifting to a MeOH medium (varying time points after shift). Top: confocal imaging. Bottom: STED imaging of the same cells as directly displayed above. Representative images, not the same cell, are shown over time. (B–E) Confocal (B, D) and STED (C, E) images of fixed cells containing Pex3-Halo labeled with SiR dye. *H. polymorpha* cells grown in log-phase on glucose-containing medium (B, C) or grown for 16 h on MeOH medium (D, E). Fluorescence intensity profiles (left) of the yellow dashed line in the images (right). The same cell was used for confocal and STED imaging. (F) Quantification of peroxisome circumference using STED microscopy of fixed *H. polymorpha* cells. All peroxisomes measured are labeled using Pex3-Halo stained with SiR dye, with rings fitted at the peroxisomal signal. Mean circumference (nm) per time point (grown on glucose- or MeOH-containing medium) + standard deviation (SD, *dark gray*). The mean circumference between the different timepoints is significantly different (one-way ANOVA between samples); *p* = 4.8∗10^−9^. Three independent experiments were performed (biological triplicate). In each experiment all peroxisomes of >100 cells were measured. (G) Confocal image of live Pex3-GFP (left), as a control for the live-cell Pex3 labeling and STED imaging using the HaloTag (middle) or SNAP-tag (right) stained with SiR dye. All cells grown for 6 h on MeOH medium. All scale bars: 1 μm.

Confocal microscopy of formaldehyde-fixed cells producing endogenous Pex3-Halo incubated with a silicon rhodamine (SiR) dye showed peroxisomal profiles, indicating that the SiR dye specifically binds the tag (Figure 1A). When grown on glucose medium, a condition that does not require peroxisomal metabolic pathways, *H. polymorpha* cells contain a single, small peroxisome. Due to the small size of these peroxisomes, Pex3-Halo fluorescence appears as a filled spot upon confocal imaging in glucose-grown cells (Figure 1A, top row). However, STED nanoscopy of the same cells shows the peroxisome structure in much more detail (Figure 1A, bottom row), revealing the peroxisomal membrane as a clear ring, separated from the peroxisomal matrix. Where line profiles of the fluorescence intensity of confocal images only show one peak, the STED images show a well-defined peak at each of the membranes, clearly distinguishable from the peroxisomal matrix (Figures 1B and 1C).

When *H. polymorpha* utilizes MeOH as the sole carbon source, peroxisomes proliferate, as they are needed to catalyze the oxidation of MeOH and to prevent buildup of toxic compounds.^17^ Thus, upon a shift from glucose to MeOH-supplemented medium, the single, small peroxisome first increases in size and subsequently multiplies by fission, accompanied by further growth of the organelles.^35^ In the enlarged peroxisomes, patches of enhanced Pex3 concentrations along the peroxisome membrane can clearly be detected by STED (Figure 1A).

After 8–15 h of MeOH growth, multiple, large peroxisomes are present in the cells. Moreover, even at a methanol depletion stage (112h MeOH) many stained peroxisomes are still clearly visible, although Pex3-Halo is sparsely labeled and the signal a bit more dispersed (Figure 1A). When cells are ultimately dying and become completely autofluorescent (122h MeOH^36^), the labeled peroxisomes are no longer detected (Figure 1A). Unlike in confocal imaging, STED microscopy allows to distinguish each of the multiple peroxisomes from one another, enabling accurate peroxisome quantification, in all stages except for the dead cells. This is especially the case for the smallest peroxisomes, which cannot be distinguished using confocal imaging. This is underscored by the line profiles of the images containing many small peroxisomes, showing multiple peaks in case of STED, but not for the confocal image (Figures 1D and 1E). In addition, the spatial distribution of Pex3 at the peroxisomal membrane can be determined in STED, showing a non-uniform distribution on the membrane. STED is thus a valid method to determine the localization of proteins at the peroxisomal membrane and to precisely quantify the number and size of peroxisomes, allowing calculating the average peroxisome circumference as a function of their carbon source (Figure 1F). Upon shifting glucose-grown cells to MeOH-containing medium, the circumference, measured as the Pex3-Halo signal, increased during the first 8 h (0–8h MeOH; Figure 1F) when the single small peroxisome expands (until approx. 6 h) as well as during the first fission events. Subsequently, this growing peroxisome divides and thus becomes slightly smaller. This explains that on average the peroxisome circumference is the largest after 8 h of growth on MeOH (where some large peroxisomes divide while others are still growing). Moreover, cells that are grown even longer on MeOH, containing multiple peroxisomes due to division, have a smaller circumference. These data could not have been retrieved using confocal imaging.

To find optimal labeling conditions, we furthermore compared live-cell endogenous labeling of Pex3 fused to an SNAP-tag, HaloTag, or GFP (Figure 1G). No difference is seen between the patterns of the Halo- or SNAP-tagged protein, compared to Pex3-GFP, which has been used in earlier studies.^12^^,^^14^ Thus, both the HaloTag and the SNAP-tag enable specific labeling. As the HaloTag resulted in a higher labeling density per cell, this is the preferred method of labeling. A comparison of imaging on fixed and living cells showed similar peroxisome structures and Pex3 localization. From this, it can be concluded that live-cell STED imaging in yeast cells is possible without consequences for the peroxisome organization and that aldehyde fixation does not give rise to artifacts (Figures 1A and 1G).

Summarizing, we show that STED both in fixed and living yeast cells enables to determine the peroxisome membrane organization with much more detail as compared to confocal imaging. This enables to acquire important quantitative information including the exact number of peroxisomes, their size, and the distribution of Pex3 along the membrane.

### STED analysis reveals a heterologous distribution of Pex3 binding partners at the peroxisomal membrane

**Figure 2:**
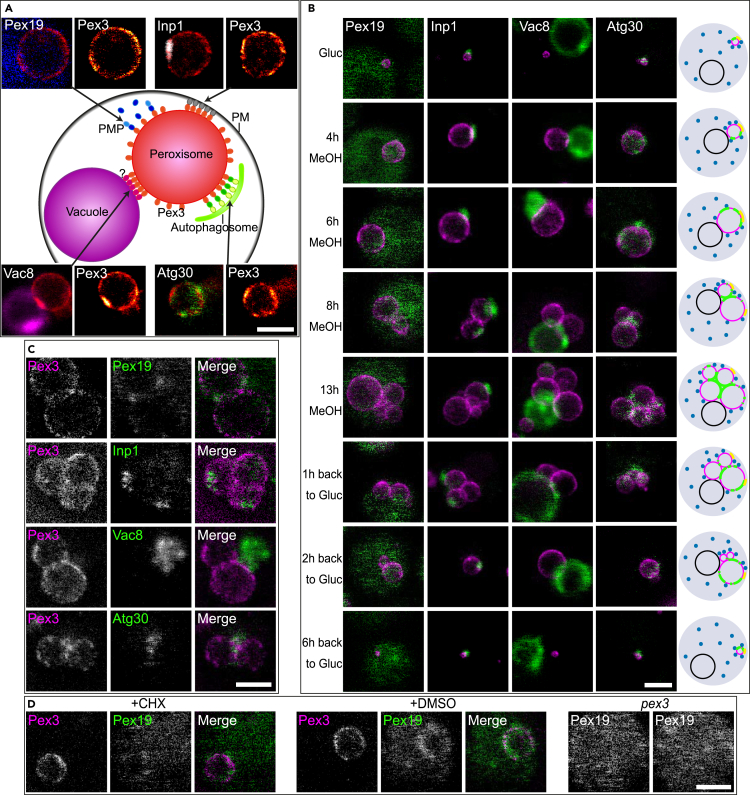
Growth-dependent localization of Pex3 and its binding partners shows unequal distribution along the peroxisomal membrane (A) Schematic depiction of the multiple functions of Pex3 (center), for simplicity all shown at a single peroxisome in one cell. PM, plasma membrane, ?, unknown vacuolar binding partner. The dual color images show fixed *H. polymorpha* cells grown for 6 h on MeOH with STED imaging of Pex3-Halo stained with SiR dye merged with confocal images of the binding partners (GFP, left panels): Pex19 (*blue*, upper left), Inp1 (*white*, upper right), vacuoles (*magenta*, lower left), and Atg30 (green, lower right). Right panels show STED of Pex3-Halo stained with SiR dye alone (red-hot), with higher intensity indicated in yellow. (B) STED images of Pex3-Halo stained with SiR dye (*magenta*) are shown in combination with confocal images of Pex19-GFP (left), Inp1-GFP (middle left), Vac8-GFP (middle right), and Atg30-GFP (right) (*green*). A model per time point (Atg30 in *green* on *magenta* peroxisomes, the vacuole in *black*, Inp1 in *yellow*, and Pex19 schematically in *blue*) is shown on the most right. Cells were pre-cultivated on glucose (stage I), transferred to MeOH medium (stage II + III), and after 13 h of growth, switched back to glucose medium (stage IV). Images were prepared from fixed cells at the indicated time points. Representative images of each condition and strain are shown. All images are the same magnification. (C) STED microscopy of fixed *H. polymorpha* cells grown for 8 h on MeOH containing Pex3-SNAP stained with SiR dye (left, *magenta*). Binding partners are tagged with Halo and stained using JF585 dye (middle, *green*). Representative STED images of Pex3 with Pex19, Inp1, Vac8, and Atg30 are shown together with the merged channel (right). (D) STED microscopy of fixed *H. polymorpha* cells grown for 8 h on MeOH containing Pex3-SNAP stained with SiR dye (*magenta*) and Pex19-Halo stained using JF585 dye (*green*) together with the merged channel. Cells are treated for 2 h with cycloheximide (CHX, left) or DMSO as a control (middle). Pex19-Halo staining with JF585 dye was also performed in *pex3* cells. Representative STED images of each experiment performed in triplicate are shown. All scale bars: 1 μm.

Using confocal imaging, Pex3-GFP patches representing relatively large peroxisome-vacuole and smaller peroxisome-plasma membrane contact sites (containing Inp1) have been identified before.^12^^,^^14^ These are also seen by STED (Figure 2A). However, STED revealed additional, tiny Pex3-GFP patches as well (Figure 1A). Next, we wanted to know whether these additional patches are related to the other, known binding partners of Pex3, namely Pex19 or Atg30, which might explain the spatial distribution of Pex3 and where it is localized on the membrane. Therefore, we performed dual color colocalization studies of Pex3 (Halo- or SNAP-tagged) with Pex19 or Atg30 (GFP- or Halo-tagged). The Halo- and SNAP-tag bound dyes are imaged using STED, and simultaneously, the GFP labels are imaged at confocal resolution. We furthermore analyzed the Pex3 distribution in relation to the localization of either Inp1-GFP or Vac8-GFP, which mark the peroxisome-plasma membrane or the peroxisome-vacuole membrane contact sites, respectively.

Our initial colocalization experiments were performed using cells grown for 6 h on methanol, as they contain one very large peroxisome, ideal to identify the protein distribution along the membrane in great detail. As shown in Figure 2A, patches where Inp1 and Pex3 accumulate overlap at peroxisome-PM contact sites and the larger Pex3 patches at peroxisome-vacuole contact sites (marked by Vac8-GFP) are evident by STED imaging, confirming previous confocal microscopy studies.^12^^,^^14^ Earlier quantification of the length of these contacts using electron microscopy revealed that these contacts are around 125 nm and 250 nm in length, respectively.^14^ Next to these two relatively large Pex3 patches, additional, tiny Pex3 patches were identified using STED nanoscopy, which could represent binding sites with Atg30 or Pex19. As shown in Figure 2A, Atg30-GFP localizes to the peroxisomal membrane in a non-homogenous manner, similar to and often colocalizing with Pex3, possibly occupying the tiny Pex3 patches where Inp1 and the vacuole do not bind. On the other hand, not all Pex3-Halo spots on the peroxisomal membrane overlap with the vacuole contact site, Inp1-GFP or Atg30-GFP, indicating that at these spots other not-yet-identified Pex3 binding partners may localize (Figure 2A). Confocal imaging revealed that Pex19-GFP does not accumulate at the peroxisomal membrane in spots, but instead is mainly present in the cytosol (Figure 2A). Therefore, no clear correlation between Pex3- and Pex19-related fluorescence is apparent.

Peroxisome formation is repressed when cells grow on glucose (here called stage I) and induced upon switching glucose-grown cells to MeOH-containing medium. In *H. polymorpha,* the single small peroxisome first grows (stage II), followed by a stage where organelles multiply, while they continue to grow (stage III). When these cells are switched back to glucose, the bulk of the peroxisomes is selectively degraded by macropexophagy (stage IV).^37^ Here, we investigated whether the distribution of Pex3 and its binding partners differ at these four stages. To construct a representative comparison, cells were collected from the same culture and fixed at different time points after switching from glucose to MeOH or subsequently back to glucose-containing medium.

Independent of this stage of peroxisome biogenesis/degradation, Pex19-GFP is mainly cytosolic and homogeneously distributed, with the exception of exclusion in a few regions (which probably are organelles like the vacuole) (Figure 2B, left panels). As these images are obtained using confocal microscopy, a minor fraction of Pex19 that might be localized at the peroxisomal membrane where it binds Pex3 is not visible in these images. However, when Pex19 is imaged by STED (using Pex19-Halo and Pex3-SNAP), a portion of the cytosolic Pex19 also appears as very tiny spots on the peroxisomal membrane (Figure 2C, top). This peroxisomal localization is only visible using STED, underlining the importance of the super-resolution imaging used here. This Pex19 localized on the membrane does not correlate with Pex3. Therefore, Atg30, but not Pex19, accumulates on some of the newly detected, tiny Pex3 patches.

Similar to Pex19, also the Inp1 distribution does not change during either peroxisome biogenesis or degradation. Inp1 invariably localizes at a single location on the membrane of a peroxisome (Figure 2B, second from left panels), which represents the peroxisome-PM contact site, where Pex3 accumulates as well.^12^ Under conditions at which multiple peroxisomes are formed (stage III), Inp1 is only present at one or at most two of the peroxisomes. The distribution of Inp1 is independent of the method of tagging, as Inp1-Halo (imaged by STED) shows the same distribution as Inp1-GFP (Figures 2A and 2C).

Peroxisome-vacuole contacts, and thus the large Pex3 patches that can occur at these contacts, are absent in stage I (Figure 2B, second from right panels). However, during induction of peroxisome proliferation (stage II) the peroxisomes form a contact site with the vacuole, where Pex3 forms large patches, in line with previous findings.^14^ At stage III, when multiple peroxisomes occur, at least one peroxisome contains a large Pex3 patch at the peroxisome-vacuole contact site. After completion of pexophagy (stage IV), when only a single small peroxisome remains per cell,^24^ the vacuole contact site is absent again and the result is similar to what is observed at stage I (Figure 2B, top and bottom).

Atg30-GFP is in all stages visible on the peroxisomal membrane, yet the signal is very low in stage I. In stage I and II, when one peroxisome is present per cell, Atg30 is mainly spread over the peroxisomal membrane in a few heterogeneous spots. At stage III, Atg30 localizes between the peroxisomes. Surprisingly, upon induction of pexophagy (stage IV), when multiple peroxisomes are still present, the Atg30-GFP signal appears to move away from these locations, to the cytosolically exposed outer part of the peroxisome clusters (Figure 2B, right panels). The results of both confocal imaging of Atg30-GFP (Figure 2B) and STED imaging of Atg30-Halo (Figures 2C and S2) show a similar response of the stage-dependent Atg30 localization. Using STED imaging of the Pex3 binding partners, also the Inp1 patches were evident but not patches of Vac8 (Figure 2C). This is as expected, since Vac8 does not accumulate at the peroxisome-vacuole contact site.^38^

Our comparison of confocal and STED imaging reveals that multicolor STED can be successfully applied in yeast, where it provides much more detail compared to confocal microscopy. STED confirms the known Pex3 patches at the binding sites of the peroxisome to Inp1 and the vacuole, validating the dual-color imaging. STED imaging also reveals higher Pex3 intensities where peroxisomes are in close contact with each other (Figures 1A and 2B). Generally, two peroxisomes are <30 nm apart. Next to this, we show that Atg30 is commonly present on the peroxisomal membrane and accumulates at some of the smallest Pex3 patches, which were not detected before by confocal microscopy. This unveils the spatial distribution of Pex3 along the membrane, mainly located on contact sites with its binding partners. The localization of Atg30 shifts under pexophagy-inducing conditions. We will analyze this quantitatively and in more detail in the next section.

With regard to the Pex19 localization, in confocal imaging we mainly saw a homogeneous distribution in the cytosol (Figure 2B). The significantly enhanced resolution in STED, however, also revealed a low amount of peroxisomal membrane-bound Pex19, which accumulates in patches that do not correlate with Pex3 localization (Figure 2C). In *pex3* control cells, which lack peroxisomes, Pex19 localization is only cytosolic, confirming that a portion of the Pex19 protein localizes to peroxisomes in WT cells (Figure 2D). Interestingly, upon treatment of the cells with the protein synthesis inhibitor cycloheximide (CHX), peroxisomal localization of Pex19 is strongly reduced. This was not observed in mock-treated cells to which DMSO was added (Figure 2D). These data indicate that the peroxisomal localization of Pex19 is dependent on active PMP synthesis and transport to the peroxisome.

### Spatiotemporal changes in Atg30 distribution

**Figure 3:**
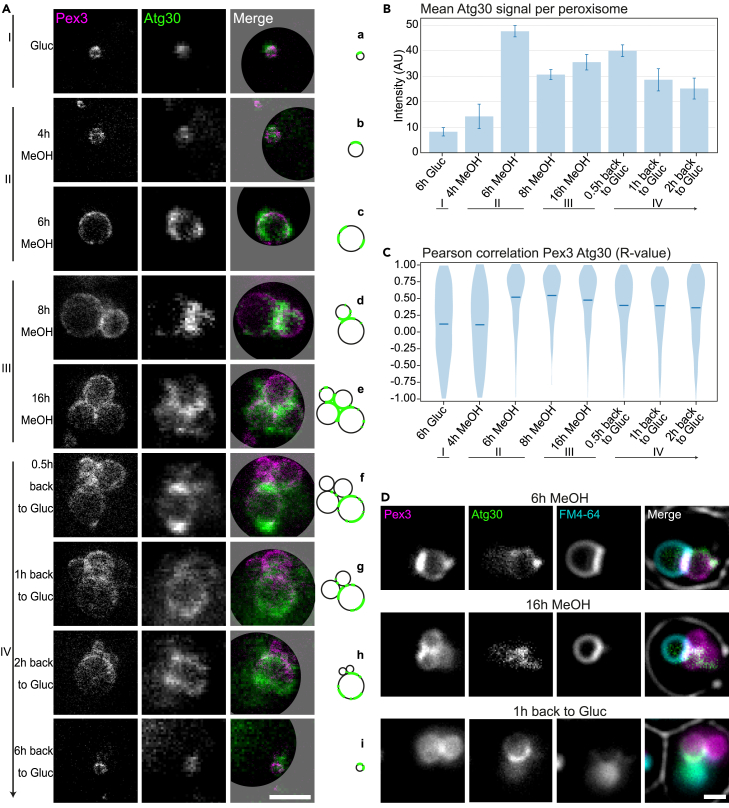
Atg30 localization shifts upon induction of pexophagy (A) STED imaging of Pex3-Halo stained with SiR dye (*magenta*) and Atg30-GFP (confocal, *green*). The images show the separate channels of Pex3 (left), Atg30 (middle), and the merged image (right). A model per time point (Atg30 depicted in green on the black peroxisomes) is shown on the most right, when switching from glucose (a) to MeOH medium for 4 h (b), 6 h (c), 8 h (d), and 13 h (e). Followed by switching back to glucose-containing medium for 0.5 h (f), 1 h (g), and 2 h (h). Representative images are shown. (B) Mean Atg30-GFP intensity per peroxisome during different growth conditions (glucose or MeOH medium) based on confocal imaging. Pex3-Halo is imaged by STED and used to detect individual peroxisomes. Same culture followed over time, Atg30-GFP signal measured on Pex3-labeled peroxisomes. The mean Atg30 signal + SD (*dark blue*) between the different time points is significantly different (one-way ANOVA); *p* = 3.2∗10^−8^. Three independent experiments were performed (biological triplicate). In each experiment, all peroxisomes of >100 cells were measured. (C) Pearson correlation coefficient (R value) of the moving average of Atg30-GFP (confocal) intensity with Pex3-Halo (STED) over time. Mean of all correlations per peroxisome is indicated in dark blue; spread of the data is shown in light blue in the violin plot. The mean correlation between the different time points is not equal (one-way ANOVA); *p* = 1.9∗10^−7^. Three independent experiments were performed (biological triplicate). In each experiment all peroxisomes of >100 cells were measured. (D) Positioning of Atg30-GFP relative to the peroxisome and vacuole. Widefield fluorescence microscopy live-cell imaging of *H. polymorpha* cells grown on MeOH medium for 6 h (top) and 16 h (middle) or when switched back to glucose medium for 1 h (bottom). Peroxisomes are labeled using Pex3-Halo with SiR dye (*magenta*), Atg30 is tagged using GFP (*green*), and vacuoles are stained with FM 4-64 dye (*cyan*). Merged image (right) shows all three colors, including the cell exterior visualized in the bright-field channel (gray). All scale bars: 1 μm.

Atg30 is known to play a role in pexophagy (stage IV). Interestingly, *H. polymorpha ATG30* is strongly upregulated in stage II, suggesting a role in peroxisome biogenesis as well.^18^ To get more insights into the role of Atg30 in peroxisome biology, we investigated when, where, and how abundant Atg30 is present at the four stages of peroxisome induction and degradation. For this, STED imaging of Pex3-Halo was used to detect individual peroxisomes and confocal imaging of Atg30-GFP to detect Atg30 (Figure 3A). Subsequently, the GFP fluorescence along the individual peroxisomes and the correlation between Pex3-Halo and Atg30-GFP fluorescence intensities were quantified (Figures 3B and 3C).

As indicated earlier, Atg30 invariably localizes on the peroxisomal membrane (Figure 3A [a–i]). When multiple peroxisomes are present, it accumulates where peroxisomes are in physical contact at the peroxisome clusters (Figure 3A [d and e]). This localization changes already within 30 min after induction of pexophagy, ultimately shifting to the opposite site of the peroxisome. In addition, most Atg30 is observed on the biggest peroxisome in the cell (Figure 3A [f–h]). These observations were confirmed by STED analysis on Atg30-Halo combined with confocal imaging of Pex3-GFP (Figure S2C). However, since the Atg30-Halo signal is very low (especially in stage I) and even with higher signals shows sparse labeling (Figures 2C and S2), the confocal images are preferred for correlation analysis.

For the pexophagy experiments, we invariably transfer cells precultivated on media containing 0.5% MeOH to media containing glucose. However, similar results were obtained when cells were precultivated in medium supplemented with 1% or 3% MeOH (Figure S3).

Quantification of the Atg30-GFP fluorescence intensities at peroxisomes revealed that the Atg30-GFP levels per peroxisome are very low and sometimes barely detectable at stage I, with only a small bright spot in some peroxisomes (Figures 3A and 3B). The signal strongly increases at stage II, in line with the observation that under these conditions *ATG30* is upregulated (four times with respect to the concentration in stage I).^18^ Peroxisomes contain the highest Atg30-GFP levels 6 h after the shift to methanol medium (mean Atg30 signal 5.8 times higher than at stage I). During pexophagy (stage IV), the Atg30 levels decrease. These data indicate that the mean Atg30-GFP intensity along the peroxisome differs significantly dependent on the growth conditions, with the highest signal at 6h MeOH growth (Figure 3B).

**Figure 4:**
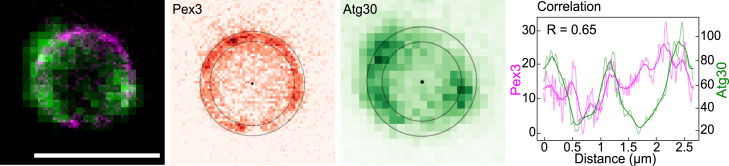
Atg30 signal and correlation with Pex3 along the peroxisomal membrane Merged image of Pex3-Halo stained with SiR dye (STED, *magenta*) and Atg30-GFP (confocal, *green*), which is used to measure the mean Pex3 and Atg30 signal intensity per peroxisome. A ring is fitted onto the peroxisomal membrane according to the Pex3 signal, along the inner and outer part of the membrane to retrieve the Pex3 signal as function of the membrane position. This same region is used to measure the Atg30 signal along the peroxisome, now using a two times wider ring (because of the larger pixel size used due to the lower resolution obtained by confocal imaging). The signal intensity is plotted along the circle length (distance). The R value of the moving average is calculated for each peroxisome (top left in the plot), to indicate its correlation. Scale bar: 1 μm.

To determine to which extent Atg30 is present at locations with elevated Pex3 levels, we performed a correlation analysis (R value). For this purpose, a ring is fitted onto the membrane of peroxisomes based on the Pex3-Halo fluorescence. Rings are localized by manually selecting the approximate center of each ring. A circle fit is applied to the peak intensities oriented from the provided center position, with an exclusion of outlier peaks (>10% deviation from the mean distance to the center). In the case of a very small ring (e.g., no clear intensity dip at the ring center), the circle is obtained by determining the center position and major axis of the segmented object at the given position (by intensity thresholding). In this way, the Pex3-Halo fluorescence signal can be plotted as a function of the membrane position (Figure 4). Similarly, Atg30-GFP fluorescence is measured and plotted on this same location. However, for this we used a two times wider line profile on the ring (because of the lower resolution of confocal imaging). To indicate the correlation, the R value of the moving average is calculated for each peroxisome (Figure 4).

At stage I (single, small peroxisome, repressed on 6 h glucose) a huge spread between positive and negative correlation values was found, with only a very weak average correlation of R = 0.15 (Figures 3C and S4A). Both this close-to-zero R value and the large spread indicate a random localization of Atg30 compared to Pex3. Thus, the often low Atg30-GFP signal does not correlate with the Pex3 localization. Shortly after induction of peroxisome proliferation, when still a single peroxisome is present per cell, both proteins show a similar distribution compared to the peroxisome-repressing condition (stage I). Again, the R values show a broad distribution with an average value close to zero (R = 0.12). Thus, the Atg30-Pex3 localizations are again uncorrelated. Yet, later in stage II (growing, single peroxisome; 6h MeOH), as well as in stage III (peroxisome multiplication, up to 16h MeOH) a clear positive correlation between the Pex3 and Atg30 localization becomes apparent. Both the distribution in the R values becomes significantly narrower and the average R value increases up to 0.52, which is considered a moderate positive relationship (Figure 3C). Thus, at growth conditions in which a larger or multiple peroxisome(s) are formed, Atg30 accumulates specifically at sites of enhanced Pex3 levels. At stage III (multiple peroxisomes), both the Pex3 and Atg30 levels are generally the highest at regions where multiple peroxisomes are in contact with each other (Figure 3A [d and e]). Also, the clear localizations of Pex3 (patches) with other organelles disappear. Thus, both Pex3 and Atg30 primarily accumulate within clusters of multiple peroxisomes at this stage, and the Atg30 signal is often, but not always, highest at dense Pex3 regions. At stage IV, 30 min after the shift to glucose medium (to induce pexophagy), the Atg30-GFP localization drastically changes. Instead of a localization at the peroxisome-peroxisome contacts, most of the Atg30 localizes to a single of the many peroxisomes (Figure 3A). Interestingly, the Pex3 localization does not make this transition, as Pex3 remains mostly at the peroxisome-peroxisome contacts. Therefore, the shifted Atg30 localization results in a decreased correlation with Pex3 (Figures 3C and S4A). Yet, since part of the Atg30 remains located at the peroxisome-peroxisome contacts and Atg30 mainly moves outward on one of the peroxisomes in the cluster, a positive correlation remains.

Although the violin plot shows a huge spread between positive and negative correlation values at stage I and onset of stage II (Figure 3C), the variation between technical replicates (SD) is low (Figure S4A). The spread is thus induced by the random localization of Atg30 compared to Pex3, whereby the Atg30 intensity is in some exceptional cases very low (for an example see Figure S4B). At all later stages under growing conditions (stage II, III; 6–16h MeOH) and initially upon induction of pexophagy (stage IV; 0.5–2h back to Gluc), a higher correlation is observed between Atg30 and Pex3.

During pexophagy, an autophagosomal membrane is formed around a single peroxisome, which fuses with the vacuole and results in peroxisome degradation. In this manner, the peroxisomes are broken down one by one.^37^ To investigate if Atg30 localizes close to the vacuole, we performed a triple-color labeling of Pex3, Atg30, and the vacuole (FM4-64). From these images, it can be concluded that in stage II Atg30 does not accumulate at the peroxisome-vacuole contact site. Instead, it mainly gathers at small peroxisomes (Figures 3D [6h MeOH] and S5A) on the opposite side. Since in stage III Atg30 localizes mainly at the peroxisome-peroxisome contacts if multiple peroxisomes are present (Figures 3D [16h MeOH] and S5B) and the peroxisome-vacuole contact site is on the outside of the cluster, Atg30 does not colocalize with vacuoles at stage III as well. Next, during pexophagy in stage IV, Atg30 relocates to the outside of one of the larger peroxisomes, which sometimes indeed leads to Atg30 localization close to the vacuole (Figure 3D; 1 h back to Gluc). However, not consistently, as sometimes Atg30 assembles on a peroxisome not in contact with the vacuole, which likely is the single peroxisome remaining in each cell after completion of the pexophagy process (Figure S5C). When visualizing the organelles using widefield microscopy (Figures 3D and S5), the contacts appear clearer than when imaged using confocal or STED microcopy, since the entire thickness of the sample (whole cell) is imaged rather than only the part in focus, middle part of the peroxisome.

In conclusion, during peroxisomal growth and multiplication (stage II, III), when Pex3 shows a clear colocalization with the vacuole, Atg30 mostly localizes at small peroxisomes or remains within peroxisome clusters and thus away from the vacuole. Since no clear Pex3 patch with the vacuole (present during peroxisomal growth and proliferation; stage II, III) is seen during pexophagy, Atg30 does not localize on the peroxisome-vacuole contact site. However, Atg30 is sometimes close to the vacuole during pexophagy (stage IV, Figure 3D), where it most likely relocalizes to another contact site (e.g., peroxisome-autophagosome contacts).

**Figure 5:**
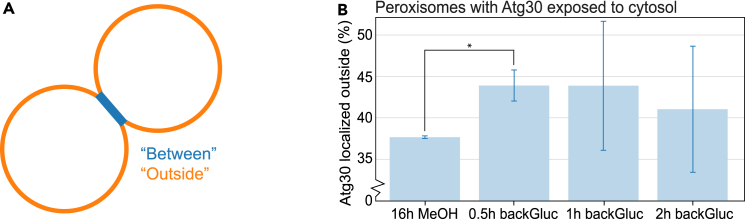
Atg30 relocalization on the peroxisomal membrane upon pexophagy induction (A) Schematic representation of the Atg30 signal separated on the Pex3-labeled peroxisomal membrane: “Between” adjacent peroxisomes at peroxisome-peroxisome contacts/inside clusters (*blue*) compared to “Outside” of the peroxisome membrane, which is not adjacent to another peroxisome and instead is exposed to the cytosol (*orange*). (B) Percentage of cells where the mean intensity of Atg30-GFP is higher at the “Outside” than “Between” peroxisomes. To determine this percentage, the mean Atg30 signal intensity “Outside” is divided by the mean intensity “Between” the peroxisomes (see A). The percentages (mean + SD, *dark blue*) are shown dependent on the growth condition, before and during induced pexophagy (MeOH or Gluc-containing medium); 16h MeOH and 0.5 h back to glucose are significantly different (one-tailed paired t-test; ∗*p* = 0.019), the other time points are not (*p* = 0.18 for 1 h back and *p* = 0.30 for 2 h back to glucose compared to 16h MeOH). Three independent experiments were performed (biological triplicate). In each experiment all peroxisomes of >100 cells were measured.

Despite the fact that Atg30 is crucial for pexophagy, the correlation of Atg30 with Pex3 is highest when peroxisomes are not degraded, but instead proliferate. Moreover, during pexophagy the Atg30 intensity and correlation with Pex3 even slightly decrease, and in addition, the Atg30 signal on the peroxisomes is not the highest upon induction of pexophagy. Instead, we observed Atg30 relocalization upon pexophagy induction, from being localized at peroxisome-peroxisome contacts, to regions exposed to the cytosol. Thus, most likely this positional shift of Atg30 is key to its function in pexophagy. To investigate this further, we quantified the relocalization. First, we separated the Atg30-GFP signal into two categories: “Between” (at the peroxisome-peroxisome contacts or inside peroxisome clusters) and “Outside” (outside of the peroxisome-peroxisome contacts/clusters) (Figure 5A). Next, the predominant localization of Atg30 per cell was determined, by the largest value of the mean Atg30-GFP signal, for which the peroxisome is classified accordingly (“Between” or “Outside”). When comparing the percentage of peroxisomes that mainly have Atg30 localized “Outside,” our results show a significant increase rapidly after pexophagy induction (stage IV, starting at 0.5 h after the switch to glucose), compared to stage III, 16h MeOH (Figure 5B).

To conclude, we show that upon inducing pexophagy, three distinct effects occur: (1) Atg30 colocalizes less with Pex3 (Figure 3C); (2) Atg30 shows a clear relocalization from the peroxisome-peroxisome contact to the more exposed outer region facing the cytosol, which occurs especially in one peroxisome at a time (Figures 3A and 5B); and (3) after an initial increase in the Atg30 intensity per peroxisome at the onset of pexophagy, it decreases later in the process (Figure 3B).

## Discussion

Here, we show that STED nanoscopy is possible in living yeast *H*. *polymorpha* cells. STED-suitable dyes are even taken up without the requirement of electroporation (as needed for STED in living *Saccharomyces cerevisiae* cells^39^), and the dyes specifically bind the genetic tags. The labeling density with the use of the HaloTag is higher than with the use of an SNAP-tag (Figures 1C and 2B showing Pex3-Halo compared to Figure 2C showing Pex3-SNAP), similar to observations in other organisms.^34^^,^^40^^,^^41^ Our super-resolution imaging and analysis of peroxisomes revealed that STED enables very accurate quantification of peroxisome size and numbers. Moreover, we showed that Pex3, a crucial protein for peroxisome biogenesis, is spatially distributed along the peroxisomal membrane and forms multiple patches. The larger ones were previously detected by confocal microscopy, at peroxisome-vacuole contact sites and peroxisome-PM contact sites.^12^^,^^14^ We now identified smaller patches, which colocalize with the pexophagy receptor protein Atg30. We also showed that a portion of the cellular Pex19 accumulates at the peroxisomal membrane.

**Figure 6:**
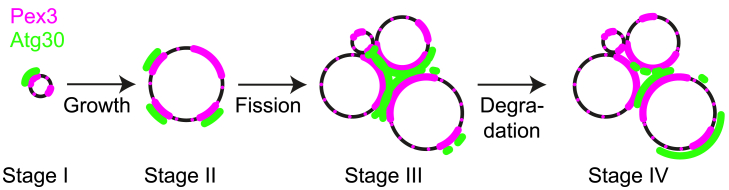
Model of Pex3 and Atg30 distribution on the peroxisomal membrane during different stages Schematic overview of Pex3 (*magenta*) and Atg30 (*green*) localization on peroxisomes (*black*), at peroxisome repressing conditions (grown on glucose; stage I) and after peroxisome induction (upon switching cells to MeOH containing medium). After inducing peroxisome growth in *H. polymorpha,* the single small peroxisome first grows (stage II) and then multiplies by fission (stage III). When these cells are switched back to glucose, the bulk of the peroxisomes are selectively degraded by pexophagy (stage IV). The Atg30 distribution along the membrane strongly depends on the growth phase. Under peroxisome repressing conditions, and immediately after growth initiation (stage I & II), Atg30 forms small clusters, colocalized with part of the Pex3 clusters. After peroxisome fission, when peroxisome-peroxisome contact sites arise, Atg30 mainly localizes at the regions where peroxisomes have physical contact with each other (stage III). During pexophagy, on the largest peroxisome a significant portion of the Atg30 relocalizes from the peroxisome-peroxisome contacts toward a part of the membrane that is exposed to the cytosol (stage IV). Under each of these growth conditions, Pex3 is always present over the complete peroxisomal membrane, yet not homogeneously. It forms patches of Pex3 clusters of which the localization partially corresponding with Atg30, Inp1 (at peroxisome-plasma membrane contacts) and in high amounts at peroxisome-vacuole contact sites. When multiple peroxisomes are present (stage III), Pex3 mainly accumulates at the peroxisome-peroxisome contact sites. At stage IV (peroxisome degradation), Pex3 does not form clear patches at the vacuole contact site anymore.

The great value of quantitative STED analysis using a large dataset was demonstrated by the comprehensive description that could be established of the spatiotemporal distribution of Pex3 and Atg30, at different stages of peroxisome proliferation and degradation (Figures 2B, 3A, and 6).

Although most models depict multiple processes and proteins binding Pex3 simultaneously in one scheme (Figures 2A and 2B), this is too simplified, e.g., because an individual peroxisome is not growing and degraded by pexophagy at the same time. Based on our observations, we designed a novel model in which Pex3 as well as Atg30 localization changes, dependent on the stage in the peroxisome biogenesis and degradation cycle (Figure 6).

To determine if the Pex3 distribution across the peroxisomal membrane and colocalization with its binding partners differs between growth conditions, we controlled biogenesis and degradation by changing the growth conditions of the cells (Figure 2B). Our data showed that Pex3 is located all over the peroxisomal membrane, although not in a homogeneous manner. Under all growth conditions, more intense Pex3 clusters are present, some with a higher intensity than others, especially on growing peroxisomes (Figure 1A). This can be more clearly seen by the “red-hot” staining (Figure 2A). After peroxisome fission, Pex3 still shows some patches along the membrane, but also accumulates at the peroxisome-peroxisome contacts, irrespective of pexophagy (stage III and IV).

We here studied the spatiotemporal distribution of the autophagy receptor Atg30 at various stages of peroxisome induction and pexophagy. When peroxisome formation is repressed (stage I) or proliferation is induced (stage II), Atg30 localizes at a single or multiple Pex3 spots but is not present at the peroxisome-vacuole contact site that most likely is involved in growth of the peroxisomal membrane (stage II; Figures 3A and 3D). In cells containing multiple peroxisomes, Atg30 mainly localizes at the peroxisome-peroxisome contacts, similar to Pex3 (stage III). However, during pexophagy Atg30 moves to the outside of peroxisomal clusters (Figures 3A and 5B). Our data suggest that the largest peroxisome initially associates via Atg30 with the autophagosome, which then fuses with the vacuole. During this process, the autophagosomal membranes are present in between the peroxisome and vacuole. This therefore differs from the situation at the Pex3-mediated peroxisome-vacuole contact sites, where both membranes are in direct contact.^14^ The largest peroxisome is often degraded first, which is in line with the observation that this organelle shows most Atg30 signal (Figure 3A). The smallest peroxisome contains less Atg30. Most likely, this single, small peroxisome invariably remains in each cell and is protected against pexophagy.^22^

Surprisingly, Atg30 is also present when peroxisomes are growing and pexophagy does not occur (Figure 3A [b and c]). Under peroxisome repressing conditions (stage I), little Atg30 is present on the small peroxisomes (Figure 3B), causing the localization to be relatively inaccurate here and making it hard to use high-resolution STED settings, which already show very low, sparse Atg30 signal when even more Atg30 is present (Figure S2). As the peroxisomes grow, the total amount of Atg30 also increases. At conditions of rapid peroxisome expansion (stage II, 6h MeOH), peroxisomes contain the highest levels of Atg30 per peroxisome, which even exceeds the levels obtained immediately after pexophagy induction. During peroxisomal multiplication (stage III), Atg30 distributes unequally and appears to preferably localize at small (probably younger) peroxisomes and later between them (Figure 3D). Since Atg30 probably recruits the phagophore while remaining at the peroxisome, it does not need to be close to the vacuole yet. Especially not during peroxisomal proliferation, when the vacuole is located near peroxisomes. In *P. pastoris*, Atg30 colocalizes with peroxisomes as well as with the vacuole during pexophagy.^16^ During peroxisomal proliferation, the localization of *Hp*Atg30 is similar to *Pp*Atg30. Both localize at the peroxisomal membrane, as well as in a bright spot on the other side of the vacuole contact. *Pp*Pex3-Atg30 interaction is not required to localize Atg30 to the peroxisome and is therefore targeted in a Pex3-independent way.^28^

It could be speculated that after the growth of an additional peroxisome, Atg30 stays at the peroxisomal membrane site where multiple peroxisomes are in contact with each other, when not needed for pexophagy (Figure 3A [d and e]). Hereby, the correlation of Atg30 with Pex3 is surprisingly highest, since Pex3 is also intense at the sandwiched peroxisome clusters (Figure 3C). However, it could be present in its non-phosphorylated form, whereby it might play a different yet unknown role in peroxisome proliferation. In this shielded location, Atg30 could be inaccessible and thereby protected from phosphorylation. Atg30/36 phosphorylation needs to find place in order to be activated for its function in pexophagy in both *P. pastoris* and *S. cerevisiae*.^27^^,^^42^ For this, Pex1/Pex6 indirectly interacts with *Sc*Atg36 on Pex3. The Pex1/Pex6 complex suppresses pexophagy by inhibiting Hrr25-dependent phosphorylation of Atg36. Even without assistance from other membrane factors, it regulates Atg36 by binding Pex1.^17^^,^^43^ Hence, it could be that in *H. polymorpha*, Atg30 phosphorylation is physically prevented by the tight peroxisome-peroxisome interactions at the organelle cluster. Thereby, non-phosphorylated Atg30 might have different binding properties, which changes the Atg30 pattern, or it could change due to binding of Atg30 to the autophagosome.

Upon induction of pexophagy, the peroxisome circumference and Pex3 correlation with Atg30 both are lower (Figures 1F and 3C). This mainly occurs because the peroxisomes are quickly degraded, and the Atg30 signal becomes unequally distributed over the peroxisomal membrane, shifting position and mainly focusing on one peroxisome. Surprisingly, Atg30 intensity peaks when a single peroxisome is rapidly growing (6h MeOH, Figure 3B). The ratio of Atg30 signal out of compared to at the peroxisome-peroxisome contact sites is slightly higher after switching back to glucose, whereby Atg30 is probably needed for pexophagy and might become accessible to play a role in pexophagy (Figure 5B). To bind Atg30 and recruit Atg8 or Atg11, *Pp*Pex3 competes with Atg37. When Atg37 is bound, Pex3 is displaced and Hrr25 is recruited to Atg30.^27^ This displaced Pex3 possibly not (always) colocalizes with Atg30 anymore and would result in lower correlation during pexophagy. This lower correlation is also seen here, and the difference is especially clear when comparing the average over time (with small spread between replicates; Figure S4A).

Analysis of Pex19 localization showed its constant presence in the cytosol independent of the growth conditions, where it binds newly formed PMPs (Figure 2B). STED revealed that a small portion of Pex19 is present on the peroxisomal membrane as well (Figure 2C), dependent on PMP import and the presence of peroxisomes (Figure 2D). The very small patches at which Pex19 accumulates on the membrane are only detectable using STED in *H. polymorpha* cells and hence were not described before. The Pex19 localization on the peroxisomal membranes does not coincide with the high-intensity Pex3 patches, yet free Pex3 might interact with cargo-bound Pex19 (higher affinity for Pex3 than Pex19 alone^10^). These findings are in line with immunofluorescence studies using human and mouse cells in which PEX19 was found both in the cytosol and at peroxisomal membranes as well, where most of the protein localized in the cytosol, with a varying ratio dependent on the cell type.^44^^,^^45^

Finally, we confirmed the intense clustering of Pex3 at peroxisome-PM contact sites where Pex3 colocalizes with Inp1 (at all stages) and at peroxisome-vacuole contact sites. The latter are primarily seen in stage II and are presumably not needed when peroxisomes are not growing (Figure 2B).

Inp1, Atg30, and Pex19 are all soluble proteins. However, Inp1 and Atg30 are associated with the membrane, probably because they always bind Pex3. Pex19 is mainly located in the cytosol, and when newly formed PMPs bind, probably locates to Pex3 not bound to Inp1 yet, but might be associated with Atg30 on a different Pex3 binding site.^28^ *Sc*Pex19 and Inp1 share a conserved LxxLL motif, with which they compete for binding Pex3 at the same site. Thereby, association of Inp1 prevents Pex3 from binding to Pex19.^13^

Here, we present a complete overview of the Pex3 localization during peroxisomal biogenesis and degradation, together with its binding partners. This highlights the use of STED, specific localization of Atg30, and its shift dependent on the carbon source. We use Atg30 as an example to precisely localize and quantify data with STED. The question remains how Pex3 can distinguish between different proteins to favor one process over the other, whereby it might accumulate or stay (stuck or due to higher affinity) when highly needed or bound. Pexophagy might thereby play a role in preserving the peroxisome population when Atg30 is exposed, in harmony with the important role of Pex3 in peroxisomal biogenesis. Together, they could maintain cell viability and regulate the healthy balance between proliferation and degradation.

### Limitations of the study

Here, we show that STED nanoscopy has major advantages over the conventional fluorescence microscopy methods, generally used in peroxisome research. However, there are still a few limitations. For instance, STED does not allow to determine the distance between different, small Pex3 proteins/patches on the membrane of a single peroxisome. Initial autocorrelation analysis of Pex3 alone did not indicate a certain pattern or correlation (because it is rather correlated with its binding partners than on a fixed position on the membrane). To accurately measure the distance between organelles, STED could be combined with correlative light and electron microscopy. To determine an even more exact localization of Pex3 and its binding partners, fluorescent techniques such as MINFLUX or MINSTED could be applied to improve the resolution.

Even though the yeast species *H. polymorpha* and *P. pastoris* are highly conserved, there are several differences in the molecular mechanisms of pexophagy. For instance, when glucose is used to induce peroxisome degradation in *H. polymorpha*, the peroxisomes are degraded by macropexophagy. However, at the same experimental conditions peroxisomes are degraded by micropexophagy in *P. pastoris*. This limits the possibilities to translate the information obtained in research on *P. pastoris* to *H. polymorpha*. In *S. cerevisiae*, the pexophagy receptor Atg36 also associates with Pex3; however, although its function is similar to HpAtg30 and PpAtg30, these Atg proteins do not share any sequence homology.

Next to this, the overview of Pex3 with its binding partners as shown here could still be incomplete, since there might be other, yet unknown binding partners of Pex3 to be revealed in the future.

## STAR★Methods

### Key resources table

**Table:** 

REAGENT or RESOURCE	SOURCE	IDENTIFIER
**Bacterial and virus strains**		

*E. coli* DH5α	Hanahan et al.^46^	N/A

**Chemicals, peptides, and recombinant proteins**		

Cycloheximide	Sigma-Aldrich	Cat#C7698
Dimethyl sulfoxide (DMSO)	Boom B.V.	CAS#67-68-5
SiR-Halo, JF585-Halo	Provided by A. N. Butkevich	N/A
SNAP-Cell 647-SiR	New England Biolabs	Cat#S9102S
FM 4-64 Dye (N-(3-Triethylammoniumpropyl)-4-(6-(4-(Diethylamino) Phenyl) Hexatrienyl) Pyridinium Dibromide)	Invitrogen	Cat#T13320

**Deposited data**		

All raw data is publicly available via DataverseNL	This study	DataverseNL: https://doi.org/10.34894/FNUHXH

**Experimental models: organisms/strains**		

*H. polymorpha* yku80: NCYC495 *YKU80::URA3*, leu1.1	Saraya et al.^47^	N/A
*H. polymorpha* P_PEX3_ Pex3-Halo: *yku80* with integration of plasmid pHIPZ_Pex3_Halo; leu1.1, Zeo^R^	This study	N/A
*H. polymorpha* P_PEX3_ Pex3-GFP: *yku80* with integration of plasmid pSEM61; leu1.1, Zeo^R^	This study	N/A
*H. polymorpha* P_PEX3_ Pex3-SNAP: *yku80* with integration of plasmid pHIPZ_Pex3_SNAP; leu1.1, Zeo^R^	This study	N/A
*H. polymorpha* P_PEX3_ Pex3-SNAP: *yku80* with integration of plasmid pHIPN_Pex3_SNAP; leu1.1, Nat^R^	This study	N/A
*H. polymorpha* P_PEX3_ Pex3-SNAP P_VAC8_ Vac8-Halo: *yku80* Pex3_SNAP with integration of plasmid pHIPZ_Vac8_Halo; leu1.1, Zeo^R^, Nat^R^	This study	N/A
*H. polymorpha* P_PEX3_ Pex3-SNAP P_ATG30_ Atg30-Halo: *yku80* Pex3_SNAP with integration of plasmid pHIPZ_Atg30_Halo; leu1.1, Zeo^R^, Nat^R^	This study	N/A
*H. polymorpha* P_PEX3_ Pex3-SNAP P_INP1_ Inp1-Halo: *yku80* Pex3_SNAP with integration of plasmid pHIPZ_Inp1_Halo; leu1.1, Zeo^R^, Nat^R^	This study	N/A
*H. polymorpha* P_PEX3_ Pex3-SNAP P_PEX19_ Pex19-Halo: *yku80* Pex3_SNAP with integration of plasmid pHIPZ_Pex19_Halo; leu1.1, Zeo^R^, Nat^R^	This study	N/A
*H. polymorpha pex3* (*RBG1):* NCYC495 *PEX3::URA3, leu1.1*	Baerends et al.^48^	N/A
*H. polymorpha pex3* P_PEX19_ Pex19-Halo: *pex3* with integration of plasmid pHIPZ_Pex19_Halo; leu1.1, Zeo^R^, Nat^R^	This study	N/A
*H. polymorpha* P_PEX3_ Pex3-Halo: *yku80* with integration of plasmid pHIPN_Pex3_Halo; leu1.1, Nat^R^	This study	N/A
*H. polymorpha* P_ATG30_ Atg30-GFP: *yku80* with integration of plasmid pAMK182; leu1.1, Zeo^R^	Wu et al.^29^	N/A
*H. polymorpha* P_ATG30_ Atg30-GFP P_PEX3_ Pex3-Halo: *yku80* Atg30_GFP with integration of plasmid pHIPN_Pex3_Halo; leu1.1, Zeo^R^, Nat^R^	This study	N/A
*H. polymorpha* P_PEX19_ Pex19-GFP P_PEX3_ Pex3-Halo: *yku80* producing Pex19_mGFP with integration of plasmid pHIPN_Pex3_Halo; leu1.1, Zeo^R^, Nat^R^	This study	N/A
*H. polymorpha* P_VAC8_ Vac8-GFP P_PEX3_ Pex3-Halo: *yku80* producing Vac8_mGFP with integration of plasmid pHIPN_Pex3_Halo; leu1.1, Zeo^R^, Nat^R^	This study	N/A
*H. polymorpha* P_INP1_ Inp1-GFP P_PEX3_ Pex3-Halo: *yku80* producing pAMK6 with integration of plasmid pHIPN_Pex3_Halo; leu1.1, Zeo^R^, Nat^R^	This study	N/A

**Oligonucleotides**		

Primer Ppex3_Fw: CCTGTTGCGGCAAGATATAG	This study	N/A
Primer Halo_Rv: CTTGCAGCAGATTCAGACC	This study	N/A
Primer Snap_Rv: GAATGGGCACGGGATTTC	This study	N/A
Primer Pex19_Fw: CCATGTTTAAGCTTATGAGCGAG	This study	N/A
Primer Pex19_BamHI _Rv: GCACGCGGATCCTGTT TGTTTGCAAGTGTCTTC	This study	N/A
Primer Ppex19_Fw: CTGCTCGTCTATCTATTTAGGC	This study	N/A
Primer Pinp1_Fw: GCTCATCGCTTATGTCACC	This study	N/A
Primer Patg30_Fw: GACTTAGCACGCCTTGGCTC	This study	N/A
Primer Pvac8_Fw: GGCTACCCAGGATAAGAAC	This study	N/A

**Recombinant DNA**		

Plasmid pUC57_Halo: pUC57 plasmid containing 45bp Halo linker and HaloTag7 fragment; Amp^R^	GenScript	N/A
Plasmid pUC57_SNAP: pUC57 plasmid containing pSNAPf-tag fragment; Amp^R^	GenScript	N/A
Plasmid pSEM61: pHIPZ containing the C-terminal part of Pex3 fused to mGFP; Zeo^R^, Amp^R^	Wu et al.^14^	N/A
Plasmid pHIPZ_Pex3_Halo: pHIPZ containing the C-terminal part of Pex3 fused to HaloTag; Zeo^R^, Amp^R^	This study	N/A
Plasmid pHIPN_Pex3_Halo: pHIPN containing the C-terminal part of Pex3 fused to HaloTag; Nat^R^, Amp^R^	This study	N/A
Plasmid pHIPZ_Pex3_SNAP: pHIPZ containing the C-terminal part of Pex3 fused to SNAP-tag; Zeo^R^, Amp^R^	This study	N/A
Plasmid pHIPN_Pex3_SNAP: pHIPN containing the C-terminal part of Pex3 fused to SNAP-tag^;^ Nat^R,^ Amp^R^	This study	N/A
Plasmid pAMK6: pHIPZ containing the C-terminal part of Inp1 fused to mGFP; Zeo^R^, Amp^R^	Krikken et al.^49^	N/A
Plasmid pHIPZ_Inp1_Halo: pHIPZ containing the C-terminal part of Inp1 fused to HaloTag; Zeo^R^, Amp^R^	This study	N/A
Plasmid pHIPZ_Vac8_mGFP: pHIPZ containing the C-terminal part of Vac8 fused to mGFP; Zeo^R^, Amp^R^	Singh et al.^38^	N/A
Plasmid pAMK174: pHIPZ containing the C-terminal part of Vac8 fused to mKate2; Zeo^R^, Amp^R^	Wu et al.^50^	N/A
Plasmid pAMK178: pHIPN containing the C-terminal part of Vac8 fused to mKate2; Nat^R^, Amp^R^	Wu et al.^50^	N/A
Plasmid pHIPZ_Vac8_Halo: pHIPZ containing the C-terminal part of Vac8 fused to HaloTag; Zeo^R^, Amp^R^	This study	N/A
Plasmid pHIPZ_Pex19_mGFP: pHIPZ containing the C-terminal part of Pex19 fused to mGFP; Zeo^R^, Amp^R^	This study	N/A
Plasmid pHIPZ_Pex19_Halo: pHIPZ containing the C-terminal part of Pex19 fused to HaloTag; Zeo^R^, Amp^R^	This study	N/A
Plasmid pAMK182: pHIPZ containing the C-terminal part of Atg30 fused to mGFP; Zeo^R^, Amp^R^	Wu et al.^29^	N/A
Plasmid pHIPZ_Atg30_Halo: pHIPZ containing the C-terminal part of Atg30 fused to HaloTag; Zeo^R^, Amp^R^	This study	N/A

**Software and algorithms**		

Fiji software (ImageJ 2.14.0)	Schindelin et al.^51^	https://imagej.net/software/fiji/; RRID:SCR_002285
Original code	This study	https://doi.org/10.5281/zenodo.11047200
Python version 3.8.8	Python Software Foundation	https://www.python.org; RRID:SCR_008394

### Resource availability

#### Lead contact

Further information and requests for resources and reagents should be directed to and will be fulfilled by the lead contact, Ida J. van der Klei (i.j.van.der.klei@rug.nl).

#### Materials availability

Plasmids generated in this study are available upon request.

#### Data and code availability

• All microscopy data reported in this paper has been deposited at DataverseNL and is publicly available. The DOI is listed in the key resources table.
• All original code has been deposited at Zenodo and is publicly available. The DOI is listed in the key resources table.
• Any additional information required to reanalyze the data reported in this paper is available from the lead contact upon request.

### Experimental model and study participant details

#### Strains and growth conditions *Escherichia coli*

To select for the correct plasmids using cloning, *E*. *coli* DH5α was used. The bacteria were grown at 37°C in Luria broth (LB) medium (1% bactotrypton; 0,5% yeast extract; 0,5% NaCl) supplemented with 100 μg/ml Ampicillin (Sigma-Aldrich).

#### Strains and growth conditions *Hansenula polymorpha*

The *H. polymorpha* strains used in this study are all derived from the NCYC495, *YKU80::URA3*; leu1.1 strain^47^ and are listed in the key resources table. Yeast cells were grown at 37°C with shaking (200 rpm), for normal growth on YPD medium containing 1% yeast extract, 1% peptone, and 1% glucose. For the selection of transformants, 200 μg/ml Zeocin (Invitrogen) or 100 μg/ml Nourseothricin (Werner BioAgents) was used. To grow cells on plates, 2% agar was added to the medium. For fluorescence microscopy studies, the cultures were grown in mineral medium (MM)^52^ containing 30 μg/ml leucine, 1x vitamin solution, 0.25% ammonium sulphate and 0.5% glucose or 0.5% MeOH (with 1% or 3% MeOH as a control, when indicated). For cycloheximide treatment, cells were incubated with DMSO (control) or CHX (6 mg/ml) for 1 or 2h in MM at 37°C with shaking.

### Method details

#### Molecular techniques

Plasmids and primers used in this study are listed in the key resources table. *E. coli* chemical transformation was performed using media described by Hanahan et al.^46^ and protocol according to Inoue et al.^53^ *H. polymorpha* electrotransformation was performed as detailed in Faber et al.^54^ DNA restriction enzymes were used according to the supplier manuals (Thermo Fisher Scientific). Polymerase chain reaction (PCR) was carried out using Phusion High-Fidelity Master Mix (Thermo Fisher Scientific). All correct plasmids were confirmed by LightRun sequencing (Eurofins Genomics) and compared using Clone Manager 9 (Scientific and Educational Software). After linearization and yeast integration, the strains were checked using colony PCR with DreamTaq enzyme (Thermo Fisher Scientific) of zymolyase treated cells, and imaged using the Gel Doc XR+ System (Bio-Rad).

#### Construction of strains expressing Pex3-Halo/SNAP

To localize *H. polymorpha* Pex3, it was labeled using STED suitable dyes that covalently bind the endogenously expressed, C-terminally fused HaloTag7 or pSNAPf-tag. The HaloTag and SNAP-tag DNA sequence were synthesized by Genscript and cloned in pUC57 (pUC57_Halo/SNAP). pHIPZ_Pex3_mGFP^14^ and pUC57_Halo/SNAP were restricted using *Sal*I and *Bgl*II and ligated to form pHIPZ_Pex3_Halo/SNAP. Positive colonies were checked using *Xho*I. The plasmids were linearized with *Eco*RI and transformed into *yku80* cells. Positive integrations in yeast were tested with colony PCR using primers Ppex3_Fw and Halo/SNAP_Rv.

Pex3_Halo was also cloned into the colocalization protein strains with GFP, and Pex3_SNAP in binding partners with Halo. Since most of these strains already have Zeo resistance, pHIPN plasmids with Pex3_Halo and SNAP were made. pHIPZ_Pex3_Halo/SNAP and pHIPN_Vac8_mKate2 (pAMK178^50^) were restricted using *Hind*III and *Xba*I and ligated to form pHIPN_Pex3_Halo/SNAP. Positive colonies were checked using *Xho*I and *Nco*I respectively. The plasmids were linearized using *Eco32*I and positive integrations in yeast were confirmed using colony PCR using primers Ppex3_Fw and Halo/SNAP_Rv.

#### Construction of GFP-strains Pex3 binding-partners

For the colocalization of Pex3_Halo with its binding partners expressing C-terminally tagged GFP (all endogenous), plasmid pHIPN_Pex3_Halo was linearized and confirmed as above, and added to strains with these proteins already integrated. They contained pHIPZ_Atg30, Pex19, Vac8 and Inp1_GFP (pAMK182,^29^ yHWL0025, yRIT0066^38^ and pAMK6^49^ respectively).

For the colocalization of Pex3_SNAP with its binding partners endogenously expressing Halo, plasmids pHIPZ_Inp1/Atg30_mGFP (pAMK6/pAMK182) and pUC57_Halo were restricted using BglII and SalI. pHIPZ_Vac8_mKate2 (pAMK174) and pHIPZ_Pex3_Halo were restricted using *Hind*III and BglII. All were ligated to form pHIPZ_Inp1/ATG30/Vac8_Halo. Positive colonies were checked using XhoI, and correct plasmids were linearized using *Blp*I (Inp1/Vac8) or BsmBI (Atg30). The plasmids were integrated into the yku80 and pN_Pex3_SNAP strain.

Pex19 was amplified from pHIPZ_Pex19_GFP, with primers Pex19_Fw and Pex19_BamHI _Rv. The obtained PCR product was restricted using *Bam*HI and *Hind*III and pHIPZ_Pex3_Halo using *Hind*III and *Bgl*II. Both were ligated to form pHIPZ_Pex19_Halo and positive colonies were checked using *Xho*I. The plasmid was linearized using *Bgl*II and transformed into yku80, pN_Pex3_SNAP and *pex3* (*PEX3::URA3*^48^) strains. Positive integrations in yeast were confirmed using colony PCR with primers Pinp1/atg30/vac8/pex19_Fw and Halo_Rv.

#### Live-cell labeling of yeast cells

*H. polymorpha* cells were grown until in the stationary phase in MM/glucose at 37°C shaking (200 rpm). The culture was diluted to OD_660_=0.1 and grow until an OD >1.6. This culture was again diluted in MM/MeOH and grown for 4-16 h at 37°C shaking. The cells were stained using 1 μM dye(s) for 1h at 37°C shaking and washed 3 times with growth medium without dye. Cells were stained using 1 μM SiR-Halo, JF585-Halo (provided by A. N. Butkevich) or SNAP-Cell® 647-SiR dye (S9102S, New England Biolabs). To stain the vacuoles, cells were simultaneously incubated with 2 μM FM™ 4-64 dye (T13320, Invitrogen™) for 1h.

#### HaloTag® labeling of fixed yeast cells

*H. polymorpha* cells were grown until in the stationary phase in MM/glucose at 37°C shaking (200 rpm). The culture was diluted to OD_660_=0.1 and grow until an OD >1.6. This culture was again diluted in MM/MeOH and grown for 4-16 h at 37°C shaking. 4 OD-units of cells were harvested by centrifugation at 1818 x g for 5 min at RT. The cells were washed with PBS and fixed with 2% PFA for 30 min at RT. The cells were stained using 1 μM dye(s) for 1h at RT and washed 3 times with PBS before attaching onto PLL-coated borosilicate glass coverslips (VWR®, No. 1.5H, 18 mm ∅, Cat. no 631-1580). The cells were mounted using Mowiol on a glass microscope slide.

#### Fluorescence microscopy

Widefield fluorescence microscopy images of living cells were taken at RT using a 100x 1.30 NA Plan-Neofluar objective on the fluorescence microscope (Axioskope A1, Carl Zeiss) with Micro-Manager 1.4 software and a digital camera (Coolsnap HQ2; Photometrics). For widefield fluorescence microscopy, GFP was visualized with a 470/40 nm band pass excitation filter, a 495 nm dichroic mirror and a 525/50 nm band-pass emission filter. JF585 was visualized with a 587/25 nm band pass excitation filter, a 605 nm dichroic mirror and a 647/70 nm band-pass emission filter. FM™ 4-64 fluorescence was visualized with a 546/12 nm band-pass excitation filter, a 560 nm dichromatic mirror, and a 575-640 nm band-pass emission filter. SiR fluorescence was visualized with a 620/60 nm band-pass excitation filter, a 655 nm dichromatic mirror, and a 660 nm long-pass emission filter.

STED nanoscopy was achieved using a commercial microscope (Abberior Instruments GmbH) containing a STED laser (775 nm), and four excitation lasers (640, 561, 488 and 405 nm). The microscope is also equipped with a CoolLED pE-2 excitation system and a 100 × oil immersion objective (Olympus UPLSAPO/1.40). Image acquisition was carried out using Imspector software (v16.3, Abberior Instruments). Visualization and part of the analysis was performed using Fiji software (ImageJ 2.14.0^51^). Figures were prepared using Adobe Illustrator 2022 software.

### Quantification and statistical analysis

All experiments were performed at least in triplicates, which are generally sufficient for studies with *H. polymorpha*, since the strains are genetically very stable. All STED measurements were performed in triplicate, using automated STED measurements via a Python script, as in Mol et al.^55^ A dataset of 8,063 STED images (individual cells) was collected. No data was excluded, except for microscopy images without identifiable Pex3-rings (fluorescent signal). All centers of the peroxisomal rings (Pex3 signal) per image were selected manually, circles were fitted by a home-written script and the Pex3 correlation (R-value, moving average) with the Atg30 signal was determined. Next to this, the circumference of the peroxisomes (amount of data points on Pex3 ring), the mean Pex3 intensity and the Atg30 intensity over the whole ring or only (non)overlapping with other Pex3 rings (“Outside” versus “Between” percentage) were calculated as well.

To measure the variability and significance levels of the STED measurements, all peroxisomes of >100 cells were measured for three independent experiments. The variance of the mean peroxisomal circumference, Atg30 intensity and Pearson correlation coefficient were all tested using a one-way (single factor) analysis of variance (ANOVA). The mean “Outside” or “Between” percentages were statistically tested using a one-tailed paired T-test. For the growth curves, three independent experiments were performed. All statistical details of the experiments can be found in the figure legends. Significance was defined if the *p*-value was <0.05. Calculations were performed using Python 3.8.8 and data visualization using Microsoft Excel 2016 and Adobe Illustrator 2023.

## References

[1] Farré J., Mahalingam S.S., Proietto M., Subramani S. (2019). Peroxisome biogenesis, membrane contact sites, and quality control. EMBO Rep..

[2] Walter T., Erdmann R. (2019). Current Advances in Protein Import into Peroxisomes. Protein J..

[3] Saraya R., Veenhuis M., Van Der Klei I.J. (2010). Peroxisomes as dynamic organelles: Peroxisome abundance in yeast. FEBS J..

[4] Schrader M., Bonekamp N.A., Islinger M. (2012). Fission and proliferation of peroxisomes. Biochim. Biophys. Acta.

[5] Smith J.J., Aitchison J.D. (2013). Peroxisomes take shape. Nat. Rev. Mol. Cell Biol..

[6] Jansen R.L.M., Santana-Molina C., van den Noort M., Devos D.P., van der Klei I.J. (2021). Comparative Genomics of Peroxisome Biogenesis Proteins: Making Sense of the PEX Proteins. Front. Cell Dev. Biol..

[7] Jansen R.L.M., van der Klei I.J. (2019). The peroxisome biogenesis factors Pex3 and Pex19: multitasking proteins with disputed functions. FEBS Lett..

[8] Sato Y., Shibata H., Nakatsu T., Nakano H., Kashiwayama Y., Imanaka T., Kato H. (2010). Structural basis for docking of peroxisomal membrane protein carrier Pex19p onto its receptor Pex3p. EMBO J..

[9] Schmidt F., Treiber N., Zocher G., Bjelic S., Steinmetz M.O., Kalbacher H., Stehle T., Dodt G. (2010). Insights into peroxisome function from the structure of PEX3 in complex with a soluble fragment of PEX19. J. Biol. Chem..

[10] Pinto M.P., Grou C.P., Alencastre I.S., Oliveira M.E., Sá-Miranda C., Fransen M., Azevedo J.E. (2006). The import competence of a peroxisomal membrane protein is determined by Pex19p before the docking step. J. Biol. Chem..

[11] Knoblach B., Rachubinski R.A. (2016). How peroxisomes partition between cells. A story of yeast, mammals and filamentous fungi. Curr. Opin. Cell Biol..

[12] Krikken A.M., Wu H., de Boer R., Devos D.P., Levine T.P., van der Klei I.J. (2020). Peroxisome retention involves Inp1-dependent peroxisome-plasma membrane contact sites in yeast. J. Cell Biol..

[13] Hulmes G.E., Hutchinson J.D., Dahan N., Nuttall J.M., Allwood E.G., Ayscough K.R., Hettema E.H. (2020). The Pex3-Inp1 complex tethers yeast peroxisomes to the plasma membrane. J. Cell Biol..

[14] Wu H., de Boer R., Krikken A.M., Akşit A., Yuan W., van der Klei I.J. (2019). Peroxisome development in yeast is associated with the formation of Pex3-dependent peroxisome-vacuole contact sites. Biochim. Biophys. Acta. Mol. Cell Res..

[15] Bellu A.R., Salomons F.A., Kiel J.A.K.W., Veenhuis M., Van der Klei I.J. (2002). Removal of Pex3p is an important initial stage in selective peroxisome degradation in Hansenula polymorpha. J. Biol. Chem..

[16] Farré J.C., Manjithaya R., Mathewson R.D., Subramani S. (2008). PpAtg30 tags peroxisomes for turnover by selective autophagy. Dev. Cell.

[17] Motley A.M., Nuttall J.M., Hettema E.H. (2012). Pex3-anchored Atg36 tags peroxisomes for degradation in Saccharomyces cerevisiae. EMBO J..

[18] van Zutphen T., Baerends R.J.S., Susanna K.A., de Jong A., Kuipers O.P., Veenhuis M., van der Klei I.J. (2010). Adaptation of Hansenula polymorpha to methanol: A transcriptome analysis. BMC Genom..

[19] Dusséaux S., Wajn W.T., Liu Y., Ignea C., Kampranis S.C. (2020). Transforming yeast peroxisomes into microfactories for the efficient production of high-value isoprenoids. Proc. Natl. Acad. Sci. USA.

[20] Zhai X., Gao J., Li Y., Grininger M., Zhou Y.J. (2023). Peroxisomal metabolic coupling improves fatty alcohol production from sole methanol in yeast. Proc. Natl. Acad. Sci. USA.

[21] Zhou Y.J., Buijs N.A., Zhu Z., Gómez D.O., Boonsombuti A., Siewers V., Nielsen J. (2016). Harnessing Yeast Peroxisomes for Biosynthesis of Fatty-Acid-Derived Biofuels and Chemicals with Relieved Side-Pathway Competition. J. Am. Chem. Soc..

[22] Kiel J.A.K.W., Komduur J.A., Van Der Klei I.J., Veenhuis M. (2003). Macropexophagy in Hansenula polymorpha: Facts and views. FEBS Lett..

[23] Farré J.C., Subramani S. (2004). Peroxisome turnover by micropexophagy: An autophagy-related process. Trends Cell Biol..

[24] Leão A.N., Kiel J.A.K.W. (2003). Peroxisome homeostasis in Hansenula polymorpha. FEMS Yeast Res..

[25] Nazarko T.Y., Farré J.C., Subramani S. (2009). Peroxisome Size Provides Insights into the Function of Autophagy-related Proteins. Mol. Biol. Cell.

[26] Farré J.C., Burkenroad A., Burnett S.F., Subramani S. (2013). Phosphorylation of mitophagy and pexophagy receptors coordinates their interaction with Atg8 and Atg11. EMBO Rep..

[27] Zientara-Rytter K., Ozeki K., Nazarko T.Y., Subramani S. (2018). Pex3 and Atg37 compete to regulate the interaction between the pexophagy receptor, Atg30, and the Hrr25 kinase. Autophagy.

[28] Burnett S.F., Farré J.C., Nazarko T.Y., Subramani S. (2015). Peroxisomal Pex3 activates selective autophagy of peroxisomes via interaction with the pexophagy receptor Atg30. J. Biol. Chem..

[29] Wu H. (2020).

[30] Meguro S., Zhuang X., Kirisako H., Nakatogawa H. (2020). Pex3 confines pexophagy receptor activity of Atg36 to peroxisomes by regulating hrr25-mediated phosphorylation and proteasomal degradation. J. Biol. Chem..

[31] Galiani S., Waithe D., Reglinski K., Cruz-Zaragoza L.D., Garcia E., Clausen M.P., Schliebs W., Erdmann R., Eggeling C. (2016). Super-resolution microscopy reveals compartmentalization of peroxisomal membrane proteins. J. Biol. Chem..

[32] Soliman K., Göttfert F., Rosewich H., Thoms S., Gärtner J. (2018). Super-resolution imaging reveals the sub-diffraction phenotype of Zellweger Syndrome ghosts and wild-type peroxisomes. Sci. Rep..

[33] Los G.V., Encell L.P., McDougall M.G., Hartzell D.D., Karassina N., Zimprich C., Wood M.G., Learish R., Ohana R.F., Urh M. (2008). HaloTag: A novel protein labeling technology for cell imaging and protein analysis. ACS Chem. Biol..

[34] Keppler A., Gendreizig S., Gronemeyer T., Pick H., Vogel H., Johnsson K. (2003). A general method for the covalent labeling of fusion proteins with small molecules *in vivo*. Nat. Biotechnol..

[35] Nagotu S., Saraya R., Otzen M., Veenhuis M., van der Klei I.J. (2008). Peroxisome proliferation in Hansenula polymorpha requires Dnm1p which mediates fission but not *de novo* formation. Biochim. Biophys. Acta.

[36] Kawałek A., van der Klei I.J. (2014). At neutral ph the chronological lifespan of Hansenula polymorpha increases upon enhancing the carbon source concentrations. Microb. Cell.

[37] Veenhuis M., Douma A., Harder W., Osumi M. (1983). Degradation and turnover of peroxisomes in the yeast Hansenula polymorpha induced by selective inactivation of peroxisomal enzymes. Arch. Microbiol..

[38] Singh R., Wróblewska J., de Boer R., van der Klei I.J. (2020). Hansenula Polymorpha Vac8: A Vacuolar Membrane Protein Required for Vacuole Inheritance and Nucleus-Vacuole Junction Formation. Contact.

[39] Stagge F., Mitronova G.Y., Belov V.N., Wurm C.A., Jakobs S. (2013). Snap-CLIP- and Halo-Tag Labelling of Budding Yeast Cells. PLoS One.

[40] Wilhelm J., Kuhn S., Tarnawski M., Gotthard G., Tunnermann J., Tanzer T., Karpenko J., Mertes N., Xue L., Uhrig U. (2021). Kinetic and Structural Characterization of the Self-Labeling Protein Tags HaloTag7, SNAP-tag, and CLIP-tag. Biochemistry.

[41] Erdmann R.S., Baguley S.W., Richens J.H., Wissner R.F., Xi Z., Allgeyer E.S., Zhong S., Thompson A.D., Lowe N., Butler R. (2019). Labeling Strategies Matter for Super-Resolution Microscopy: A Comparison between HaloTags and SNAP-tags. Cell Chem. Biol..

[42] Tanaka C., Tan L.J., Mochida K., Kirisako H., Koizumi M., Asai E., Sakoh-Nakatogawa M., Ohsumi Y., Nakatogawa H. (2014). Hrr25 triggers selective autophagy-related pathways by phosphorylating receptor proteins. J. Cell Biol..

[43] Yu H., Kamber R.A., Denic V. (2022). The peroxisomal exportomer directly inhibits phosphoactivation of the pexophagy receptor Atg36 to suppress pexophagy in yeast. Elife.

[44] Sacksteder K.A., Jones J.M., South S.T., Li X., Liu Y., Gould S.J. (2000). PEX19 binds multiple peroxisomal membrane proteins, is predominantly cytoplasmic, and is required for peroxisome membrane synthesis. J. Cell Biol..

[45] Colasante C., Chen J., Ahlemeyer B., Bonilla-Martinez R., Karnati S., Baumgart-Vogt E. (2017).

[46] Hanahan D. (1983). Studies on transformation of Escherichia coli with plasmids. J. Mol. Biol..

[47] Saraya R., Krikken A.M., Kiel J.A.K.W., Baerends R.J.S., Veenhuis M., van der Klei I.J. (2012). Novel genetic tools for Hansenula polymorpha. FEMS Yeast Res..

[48] Baerends R.J.S., Faber K.N., Kram A.M., Kiel J.A.K.W., Van Der Klei I.J., Veenhuis M. (2000). A stretch of positively charged amino acids at the N terminus of Hansenula polymorpha Pex3p is involved in incorporation of the protein into the peroxisomal membrane. J. Biol. Chem..

[49] Krikken A.M., Veenhuis M., Van Der Klei I.J. (2009). Hansenula polymorpha pex11 cells are affected in peroxisome retention. FEBS J..

[50] Wu F., de Boer R., Krikken A.M., Akşit A., Bordin N., Devos D.P., van der Klei I.J. (2020). Pex24 and Pex32 are required to tether peroxisomes to the ER for organelle biogenesis, positioning and segregation in yeast. J. Cell Sci..

[51] Schindelin J., Arganda-Carreras I., Frise E., Kaynig V., Longair M., Pietzsch T., Preibisch S., Rueden C., Saalfeld S., Schmid B. (2012). Fiji: An open-source platform for biological-image analysis. Nat. Methods.

[52] Van Dijken L.P., Otto R., Harder W. (1976). Growth of Hansenula polymorpha in a methanol-limited chemostat - Physiological responses due to the involvement of methanol oxidase as a key enzyme in methanol metabolism. Arch. Microbiol..

[53] Inoue H., Nojima H., Okayama H. (1990). High efficiency transformation of Escherichia coli with plasmids. Gene.

[54] Faber K.N., Haima P., Harder W., Veenhuis M., Ab G. (1994). Highly-efficient electrotransformation of the yeast Hansenula polymorpha. Curr. Genet..

[55] Mol F.N., Vlijm R. (2022). Automated STED nanoscopy for high-throughput imaging of cellular structures. bioRxiv.

