# Supplementary material for: STED super-resolution microscopy unveils the dynamics of Atg30 on yeast Pex3-labeled peroxisomes
Source: iScience. 2024 Jul 8;27(8):110481. doi: 10.1016/j.isci.2024.110481 (PMC11326945; doi:10.1016/j.isci.2024.110481)
Supplement: Document S1. Figures S1–S5 [file mmc1.pdf]

iScience, Volume *27*

Supplemental information

STED super-resolution microscopy unveils the dynamics of Atg30 on yeast Pex3-labeled peroxisomes

Eline M.F. de Lange, Frank N. Mol, Ida J. van der Klei, and Rifka Vlijm

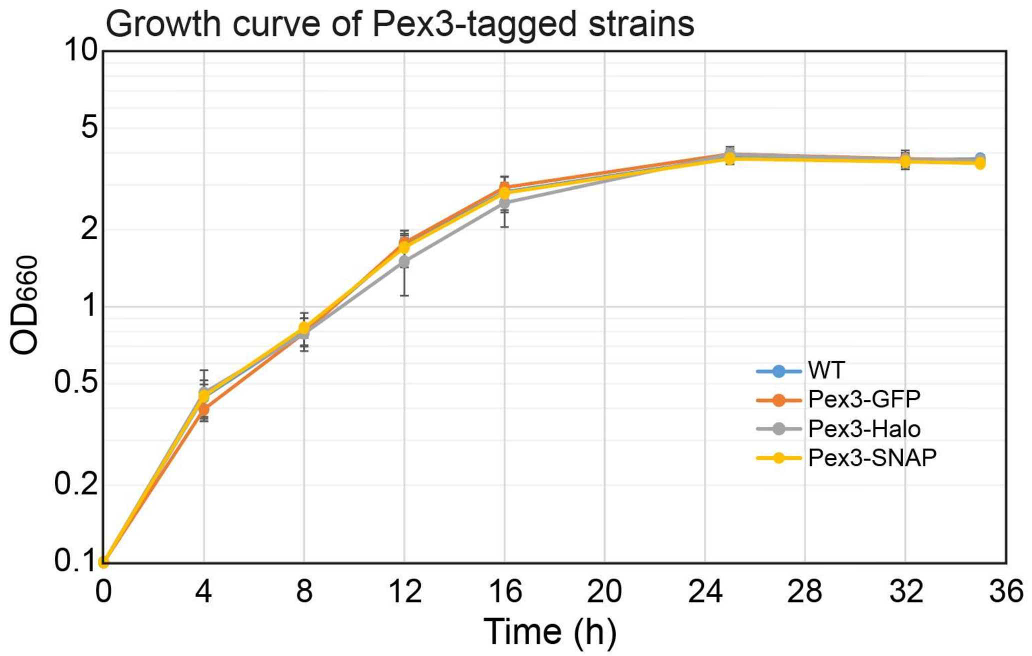

Figure S1. Cell growth of Pex3-tagged strains, related to Figure 1

Growth curve of indicated *H. polymorpha* strains in MeOH-containing medium. The optical density at 660 nm (OD_{660}, log scale) is shown over time + SD (*grey*). WT = wild type cells, n = 3 for biological replicates.

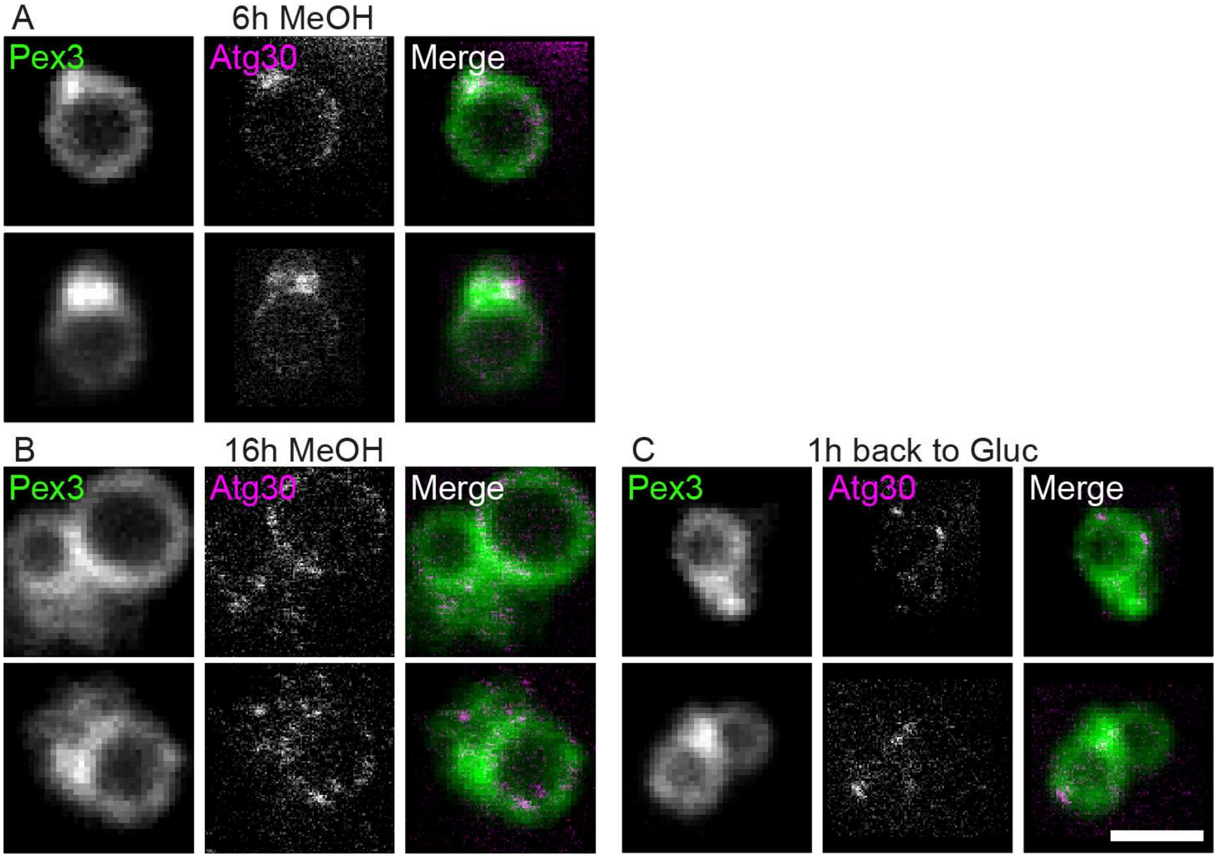

Figure S2. STED of Atg30 shows precise localization on Pex3-GFP labeled peroxisomes, related to Figure 2C

STED imaging of Atg30-Halo stained with SiR dye (*magenta*) and Pex3-GFP (confocal, *green*). *H. polymorpha* cells grown on MeOH medium for 6h (A) and 16h (B), or when switched back to glucose medium for 1h (C). The separate channels of Pex3 (left), Atg30 (middle) and the merged image (right). Scale bar 1 µm.

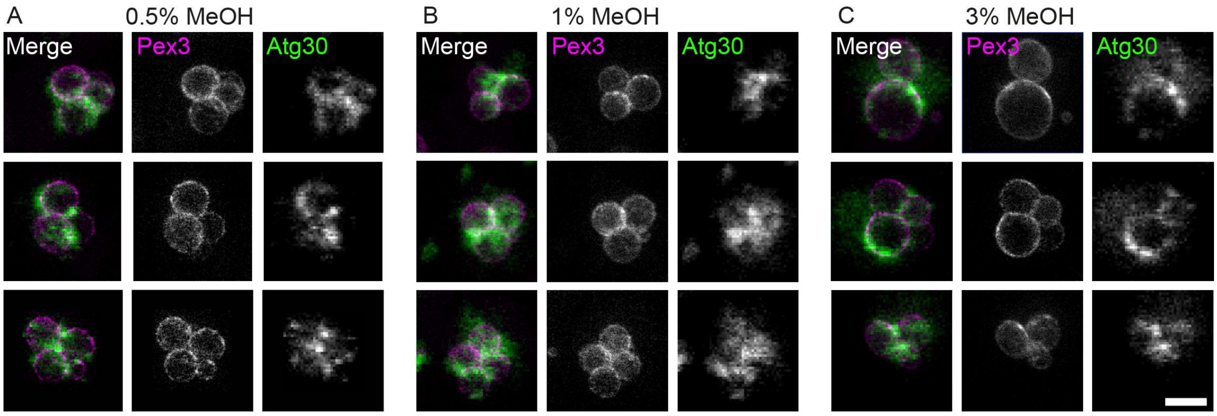

Figure S3. Different MeOH concentrations do not affect pexophagy, related to Figure 3
STED imaging of Pex3-Halo stained with SiR dye (*magenta*) and Atg30-GFP (confocal, *green*). *H. polymorpha* cells grown for 16h on medium containing 0.5% MeOH as usual (A), 1% MeOH (B) or 3% MeOH (C). 3 example images of each condition are shown, with the separate channels of Pex3 (left), Atg30 (middle) and the merged image (right). Scale bar 1 µm.

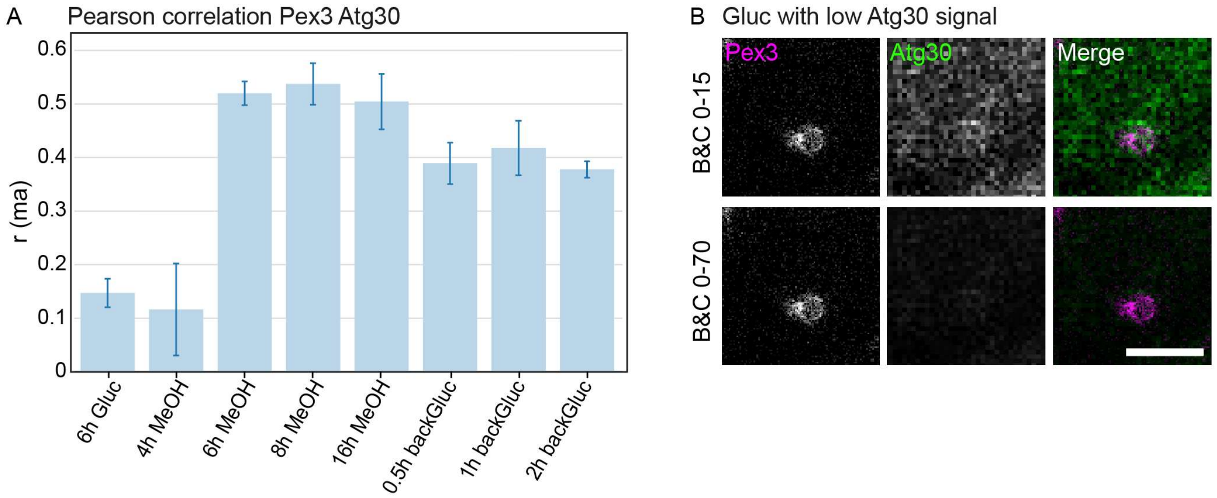

Figure S4. Pex3-Atg30 Pearson correlation and adjusted Atg30 levels, related to Figure 3
(A) Pearson correlation coefficient (r) of the moving average of Atg30 intensity with Pex3 over time. Mean intensity + SD (*dark blue*). n = 3, >100 cells per biological triplicate.
(B) STED image of Pex3-Halo stained with SiR dye (*magenta*) with confocal, low signal of Atg30-GFP (*green*). The separate channels of Pex3 (left), Atg30 (middle) and the merged image (right) are shown for a cell grown on glucose (i), similar as in Fig. 4A. Both rows show the same picture, with optimally adjusted brightness & contrast (scale 0-15 counts, upper panels) and brightness & contrast similar as in Fig. 3Aa (scale 0-70 counts, lower panels). Scale bar 1 µm.

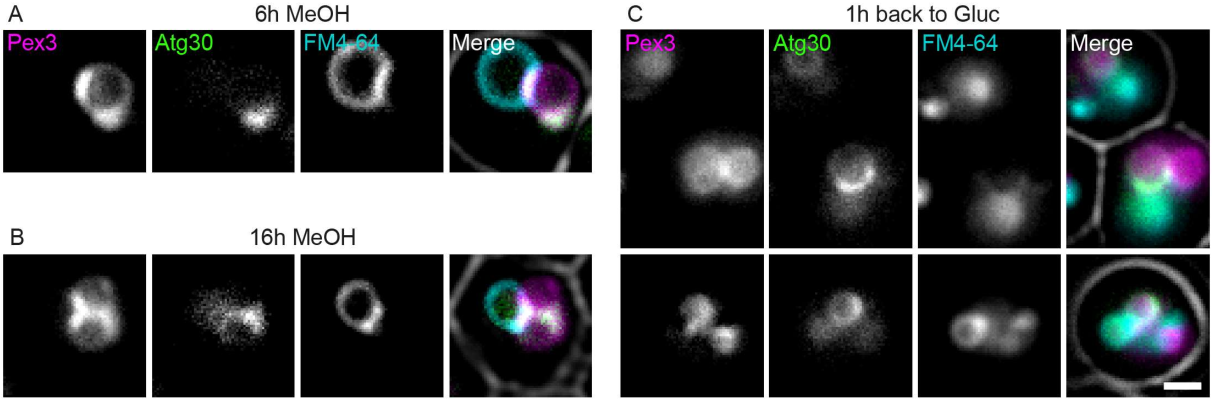

Figure S5. Atg30 location towards the peroxisome and vacuole, related to Figure 3D
Widefield fluorescence microscopy live-cell imaging of *H. polymorpha* cells grown on MeOH medium for 6h (A) and 16h (B), or when switched back to glucose medium for 1h (C). Peroxisomes are labeled using Pex3-Halo with SiR dye (*magenta*, left), Atg30 is tagged using GFP (*green*, middle left) and vacuoles are stained with FM^{TM} 4-64 dye (*cyan*, middle right). Merged image (right) shows all 3 colors, including the cell exterior visualized in the bright-field channel (gray). Scale bar 1 µm.
